# Relaxin Can Mediate Its Anti-Fibrotic Effects by Targeting the Myofibroblast NLRP3 Inflammasome at the Level of Caspase-1

**DOI:** 10.3389/fphar.2020.01201

**Published:** 2020-08-04

**Authors:** Anita A. Pinar, Alexander Yuferov, Tracey A. Gaspari, Chrishan S. Samuel

**Affiliations:** ^1^Cardiovascular Disease Program, Monash Biomedicine Discovery Institute and Department of Pharmacology, Monash University, Clayton, VIC, Australia; ^2^Department of Biochemistry and Molecular Biology, The University of Melbourne, Parkville, VIC, Australia

**Keywords:** fibrosis, myofibroblasts, NLRP3 inflammasome, caspase-1, relaxin

## Abstract

**Introduction:**

The NLRP3 inflammasome produces interleukin (IL)-1β and IL-18, which when chronically activated by transforming growth factor (TGF)-β1, contribute to fibrosis. The recombinant form of the anti-fibrotic hormone, relaxin (RLX), suppresses the pro-fibrotic influence of TGF-β1 and toll-like receptor (TLR)-4 on NLRP3 inflammasome priming and activity in human cardiac myofibroblasts and mice with cardiomyopathy. However, whether RLX also modulates components of the myofibroblast NLRP3 inflammasome remains unknown.

**Methods and Results:**

Stimulation of a human dermal fibroblast (HDF) cell line with TGF-β1 [5 ng/ml; to promote myofibroblast (HDMF) differentiation], LPS (100 ng/ml; to prime the NLRP3 inflammasome) and ATP (5 mM; to activate the NLPR3 inflammasome) (T+L+A) significantly increased NLRP3 inflammasome priming and activity after 8 and 72 h; and α-SMA expression (myofibroblast differentiation) and collagen-I deposition after 72 h. siRNA-induced knock-down of NLRP3 inflammasome priming components (NLRP3, ASC, caspase-1) in T+L+A-stimulated HDMFs for 24 h, completely knocked-down each component after 72 h. RLX (100 ng/ml) administration to T+L+A-stimulated HDMFs after control, NLRP3 or ASC siRNA transfection, equivalently suppressed IL-1β, pro-IL-18, α-SMA, and collagen-I protein levels (by 40%–50%; all p<0.05 vs. T+L+A) after 72 h, as determined by Western blotting. These RLX-induced effects were abrogated by siRNA knock-down of caspase-1.

**Conclusion:**

The anti-fibrotic actions of RLX appear to require modulation of caspase-1 within the myofibroblast NLRP3 inflammasome.

## Introduction

Inflammation is initiated by the innate immune system as part of a natural wound healing response to tissue injury. The infiltration of activated innate immune cells (Wynn, 2008) and their ability to secrete pro-inflammatory and pro-fibrotic cytokines and growth factors such as transforming growth factor (TGF)-β1 and several interleukins (ILs) including IL-1β and IL-18 are initiated to facilitate wound healing. However, when these factors are over-activated, they eventually contribute to fibrosis (Kolb et al., 2001; Gasse et al., 2007). These pro-fibrotic cytokines stimulate extracellular matrix (ECM)-producing fibroblasts to proliferate and differentiate into activated myofibroblasts (Wynn, 2008). These chronically activated myofibroblasts interact with the ECM and deposit large amounts of ECM proteins, predominantly collagens, to facilitate wound healing. However, the over-production of ECM deposition contributes to anomalous tissue remodeling and scarring that displaces the integrity of the healthy tissue (Wynn and Ramalingam, 2012; Schroer and Merryman, 2015; Li et al., 2018; Frangogiannis, 2019; Humeres and Frangogiannis, 2019).

The nucleotide-binding oligomerization domain, leucine-rich repeat and pyrin domain-containing protein 3 (NLRP3) inflammasome is a large multiprotein complex (Mangan et al., 2018; Zhou et al., 2018) that is expressed by several cell types including macrophages (Rodriguez-Alcazar et al., 2019), epithelial cells, microvascular endothelial cells and myofibroblasts (Boza et al., 2016). The NLRP3 inflammasome is formed to mediate the wound healing response to tissue injury *via* the production of IL-1β and IL-18. However, when chronically activated, it has emerged as a key contributor to fibrosis progression (Kolb et al., 2001). The NLRP3 inflammasome consists of the sensor molecule NLRP3, the adaptor protein apoptosis-associated speck-like protein containing a c-terminal caspase-recruitment domain (ASC), and the effector protease, caspase-1. The maturation and release of IL-1β is instigated *via* a two-step signaling process. The first “priming signal” synthesizes pro-IL-1β and pro-IL-18 and upregulates the expression of NLRP3 inflammasome components including NLRP3, ASC and pro-caspase-1. The second “activation signal” assembles the components of the NLRP3 inflammasome into the tri-partite protein complex, resulting in its activation, caspase-1 activation, and subsequent maturation and secretion of IL-1β and IL-18. TGF-β1 and toll-like receptor (TLR)-4 are known inducers of the NLRP3 inflammasome in myofibroblasts (Boza et al., 2016), while lipopolysaccharide (LPS) and adenosine triphosphate (ATP) can be used experimentally to prime and activate the inflammasome, respectively (Boza et al., 2016; Cáceres et al., 2019). Currently, there is a lack of available therapies that directly target scar tissue accumulation in damaged organs. Accordingly, a better understanding of the molecular mechanism involved in myofibroblast NLRP3 inflammasome-induced fibrosis progression may lead to improved and effective therapeutic targeting of NLRP3 inflammasome activation to treat fibrosis-induced end-organ damage and dysfunction.

The recombinant form of the peptide hormone relaxin, serelaxin (RLX), has emerged as a rapidly-acting anti-fibrotic therapy (Nistri et al., 2007; Du et al., 2010; Samuel et al., 2017), signaling *via* its cognate G protein-coupled receptor, relaxin family peptide receptor (RXFP)1 (Bathgate et al., 2013). Several lines of evidence have indirectly suggested that RLX may also inhibit the NLRP3 inflammasome to mediate its anti-fibrotic actions. First, RLX can suppress the pro-fibrotic influence of known inducers of myofibroblast NLPR3 inflammasome activity, including TGF-β1 (Unemori and Amento, 1990; Mookerjee et al., 2009; Sarwar et al., 2015; Wang et al., 2016) and TLR-4 (Chen et al., 2017). RLX has been found to inhibit TGF-β1 signal transduction at the level of intracellular Smad2, either through direct effects on cyclic guanosine monophosphate (cGMP) (Kocan et al., 2017) or by signaling through a RXFP1-extracellular signal-regulated kinases (ERK)-1/2 phosphorylation-neuronal nitric oxide (NO) synthase (nNOS)-NO-soluble guanylyl cyclase (sGC)-cGMP-dependent pathway (Mookerjee et al., 2009; Sarwar et al., 2015; Wang et al., 2016). This in turn results in the suppression of TGF-β1-induced myofibroblast differentiation and myofibroblast-induced collagen production (the basis of fibrosis). Second, RLX has also been found to suppress both TGF-β1- and IL-1β-mediated collagen synthesis and secretion in human dermal myofibroblasts (Unemori and Amento, 1990). Third, RLX was shown to suppress the pro-inflammatory and/or pro-fibrotic influence of IL-1β and IL-18 *in vivo*, in experimental models of renal ischemia/reperfusion injury (Collino et al., 2013) and atrial fibrillation (Beiert et al., 2017). Finally, RLX was found to attenuate myocardial ischemia/reperfusion injury by inhibiting caspase-1 activity (Valle Raleigh et al., 2017). However, caspase-1 activity can be modulated by several inflammasomes and no direct evidence was shown to link the findings obtained to the NLRP3 inflammasome in the latter study (Valle Raleigh et al., 2017).

We recently demonstrated in TGF-β1-stimulated human cardiac myofibroblasts *in vitro* and in the left ventricle of mice subjected to isoproterenol-induced cardiomyopathy *in vivo* that RLX could inhibit the TGF-β1/IL-1β/IL-18 axis *via* a RXFP1-nNOS-TLR-4-NLRP3 inflammasome-dependent mechanism (Cáceres et al., 2019). While these findings confirmed for the first time that RLX could target inducers (TGF-β1 and TLR-4) of the myofibroblast NLRP3 inflammasome to mediate its anti-fibrotic actions, it was unclear whether RLX could also modulate the NLPR3 inflammasome itself to inhibit myofibroblast differentiation and collagen-I deposition. Hence, in this study, we extended our recent findings to further delineate whether RLX also modulated the myofibroblast NLRP3 inflammasome to mediate its anti-fibrotic actions on myofibroblast differentiation and collagen I deposition. This was addressed using a RXFP1-expressing (Hossain et al., 2011; Chow et al., 2012) human dermal fibroblast (HDF) cell line subjected to siRNA-induced knock-down of specific components of NLRP3 inflammasome priming, NLRP3, ASC, or caspase-1.

## Materials and Methods

### Materials

The BJ3 human dermal fibroblast (HDF) cell line used in this study was kindly provided by William C. Hahn (Department of Medical Oncology, Dana-Farber Cancer Institute, Boston, MA). Recombinant human TGF-β1 was obtained from In Vitro Technologies (#240-B, Minneapolis, MN, United States). Recombinant human gene-2 (H2) relaxin was generously provided by Corthera Inc (San Mateo, CA, United States; a subsidiary of Novartis International AG, Basel, Switzerland). LPS (#L2630) and ATP (#A6419) were both obtained from Sigma-Aldrich (Vic, Australia).

### Culture of HDFs

BJ3 HDFs were characterized as described before (Chow et al., 2012) and cultured in DMEM medium containing 17% medium-199, 15% fetal bovine serum (FBS), 1% penicillin (50 U/ml)/streptomycin (50 μg/ml), 1% L-glutamine (200 mM) and 2.2% HEPES buffer (all obtained from Life Technologies, Mulgrave, Vic, Australia). HDFs were cultured in T-175 cm^2^ flasks at 37°C, passaged when 80%–90% confluent, and used between passages 10–14 for all of the outlined studies. At these passages, these cells were shown to express RXFP1 (Chow et al., 2012).

### Treatment of WT Human Dermal Myofibroblasts (HDMFs)

Wild-type (WT) BJ3 HDFs were seeded at a density of 1-1.5x10^5^ cells/well in 12-well plates and treated with TGF-β1 (T, 5 ng/ml) to stimulate the differentiation of these cells into myofibroblasts (HDMFs). Sub-groups of T-stimulated HDMFs were also treated with the NLRP3 inflammasome primer, LPS (L, 100 ng/ml) and activator, ATP (A, 5 mM) (T+L+A) to ensure activation of the inflammasome on myofibroblasts. As a control, separate sub-groups of cells were stimulated with L+A (without T), to determine the effects of L+A stimulation on HDFs.

Sub-groups of T- or T+L+A-treated HDMFs were then either left untreated or treated with RLX (RLX or R, 16.8 nM, which is equivalent to 100 ng/ml) for 8* h* [when NLRP3 inflammasome activation is optimally measured (Boza et al., 2016)] or for 72* h* [when changes in TGF-β1-induced myofibroblast differentiation and collagen I deposition could be measured (Samuel et al., 2004)]. The doses of each factor used were previously reported in studies that induced expression of the NLRP3 inflammasome (Boza et al., 2016) and determined the therapeutic effects of RLX on the myofibroblast NLRP3 inflammasome (Cáceres et al., 2019). Measures of NLRP3 priming [NLRP3, ASC and (pro)-caspase-1] and activity (IL-1β and IL-18) were detected after 8* h*, while changes in NLRP3 priming, myofibroblast differentiation (α-SMA expression), and collagen I deposition (as measures of fibrosis) were detected following 72* h* in T-, T+R-, T+L+A-, and T+L+A+R-treated HDMFs. These studies were conducted 6–8 separate times in duplicate.

### HDMF siRNA Transfection Studies

BJ3 HDMFs were seeded at a density of 1–1.5x10^5^ cells/well in 12-well plates, as described above. Short-interfering-RNA (siRNA) knock-down technology was used to downregulate the expression of specific components of NLRP3 inflammasome priming, compared to control siRNA, in transfected cells. Lipofectamine RNAiMax transfection reagent (#13778030; Life Technologies), Opti-MEM reduced serum medium (#11058021; Life Technologies) and four separate individual siRNAs—one control siRNA (#sc-37007), and three siRNAs each targeting NLRP3 (#sc-45469), ASC (#37281), or caspase-1 (#29235) were prepared separately (all purchased from Santa Cruz Biotechnology, Dallas, TX, United States). Lipofectamine RNAiMax was diluted 1:100 in Opti-MEM reduced serum medium and left to incubate for 30 mins at room temperature (RT). Each of the siRNAs were also individually diluted 1:100 in Opti-MEM medium in separate tubes and left to incubate for 30* min* at RT. The Lipofectamine RNAiMax/Opti-MEM mixture was then split into separate tubes and the siRNA/Opti-MEM mixtures were added to the Lipofectamine RNAiMax/Opti-MEM mixtures. These were incubated for an additional 30* min* prior to transfecting individual wells of HDFs to induce lipofectamine-mediated siRNA-knock-down of NLRP3, ASC or caspase-1 for 24* h*. Separate wells of each plate were also treated with the CTL siRNA for the same time-period. The following day, the transfection medium was replaced with media and HDFs were left to rest for an additional day. The following day, transfected HDFs were treated in duplicate, as detailed below. In one set of experiments, HDFs were transfected with the CTL siRNA for 24* h* before sub-groups of cells were further treated with T+L+A or T+L+A+R for 72* h*. This was conducted to confirm that the effects RLX on measures of NLRP3 priming [NLRP3, ASC, and (pro)-caspase-1] and activity (IL-1β and IL-18) as well as fibrosis (α-SMA and collagen I) in cells transfected with the CTL siRNA reflected that observed following corresponding treatment of untransfected WT HDMFs. These studies were conducted 4–6 separate times in duplicate. In a separate set of experiments, HDFs separately transfected with the control or individual siRNAs that targeted NLRP3, ASC, or caspase-1, were treated with T or T+L+A for 8* h* or 72* h*, to confirm knock-down of the individual NLRP3 inflammasome components. The siRNA-induced knock-down efficiency of each siRNA was determined by measuring the protein expression levels of NLRP3, ASC and (pro)-caspase-1, respectively. These studies were conducted 4–6 separate times in duplicate. In a final set of experiments, HDFs individually transfected with NLRP3 siRNA, ASC siRNA, caspase-1 siRNA or control siRNA for 24* h*, were then treated with T+L+A or T+L+A+R for 72* h* to determine which component(s) of the myofibroblast NLRP3 inflammasome RLX required an interaction with to mediate its inhibitory effects on myofibroblast differentiation and collagen I deposition. These studies were conducted 4–6 separate times in duplicate.

### Immunocytochemistry

BJ3 HDFs (1-1.5x10^6^/well) were plated and grown on poly-D-lysine-coated coverslips in 12-well plates for 24* h*. BJ3 HDFs were then treated with T+L+A in the absence or presence of RLX for 8* h* (to study the extent of NLRP3 colocalisation with caspase-1), and separately, for 72* h* (to induce expression of α-SMA, as a measure of myofibroblast differentiation). At the end of treatment, in each case, BJ3 HDMFs were washed once with warmed PBS, fixed for 10* min* in 4% PFA, and washed once again in warmed PBS, prior to staining. Treated BJ3 HDMFs were then blocked with 10% normal goat serum containing 0.2% triton X-100 for 30* min*, and then incubated overnight in either goat polyclonal anti-NLRP3 (ab4207; 1:500 dilution; Abcam, Vic, Australia), rabbit polyclonal anti-caspase-1 (ab1872; 1:750 dilution; Abcam, Vic, Australia), or rabbit polyclonal anti-α-SMA (ab5694; 1:500 dilution; Abcam, Vic, Australia) primary antibodies. The following day, primary antibodies were then aspirated and treated BJ3 HDMFs were washed 3 times in warmed PBS prior to incubation in the appropriate Alexa Fluor 488 or 594 secondary antibody (Alexa Fluor 488 donkey anti-goat IgG; #A11012; 1:1000 dilution; or Alexa Fluor 594 goat anti-rabbit IgG; #A20185; 1:750 dilution; both purchased from Life Technologies, Vic, Australia). Antibody-stained BJ3 HDMFs were then washed 3 times in warmed PBS, and cells mounted with a drop of VECTASHIELD HardSet*™* Mounting Medium containing DAPI to preserve fluorescence (Vector Laboratories; Meadowbrook, Qld, Australia) on each coverslip, following mounting of another coverslip and left to dry. T+L+A-treated BJ3 HDMFs dual stained for NLRP3 and caspase-1 were assessed for the degree of NLRP3 and caspase-1 co-localisation compared to T+L+A+R-treated BJ3 HDMFs in α-SMA differentiated HDMFs. All stained slides were scanned by Monash Micro Imaging using a Leica DMi8 Dexter microscope, at 40x magnification. Five to six field of view were randomly captured and analyzed using ImageJ Software (ImageJ, United States) to examine differences in the extent of NLRP3 and caspase-1 co-localization in T+L+A- vs. T+L+A+R-treated HDMFs.

### Western Blotting

Following the various treatment groups examined in wild-type HDMFs, as well as in cells transfected with the CTL or individual siRNAs to NLRP3, ASC or caspase-1, the media was removed and the cell layer from treatment groups were lifted and collected *via* incubation in accutase solution (500 μl per well; Sigma-Aldrich, Castle Hill, NSW, Australia) for 10 mins at 37°C. Protein was extracted from the cell layer in 20 μl of 1x RIPA lysis buffer, prepared from supplementing a 10x RIPA buffer (#9806) with PMSF (#8553), phosphatase*/*protease inhibitor (#5872) (all purchased from Cell Signalling, Danvers, MA, United States), and double-distilled water. The suspension was left to incubate in ice for 1* h*, followed by centrifugation at 1500 rpm for 10* min* at 4°C to pellet any cellular debris, leaving protein in solution. The supernatant containing the protein was then transferred to a separate Eppendorf tube, which was subsequently stored at -20°C until required for use. Equivalent volumes of protein from HDMF-cell layers were electrophoresed on mini-protean 4%–15% precast gels and analyzed by Western blotting using monoclonal antibodies to NLRP3 (#15101; 1:1000 dilution; Cell Signaling Technology, Danvers, MA, United States), ASC (ab155970; 1:1000 dilution; Abcam, Cambridge, MA, United States), pro-caspase-1 (#3866; 1:1000 dilution; Cell Signaling Technology), pro-IL-1β (MAB601-100; 1:1000 dilution; In Vitro Technologies, Noble Park North, Vic, Australia), pro-IL-18 (D043-3; 1:1000 dilution; In Vitro Technologies), and α-SMA (M0851; 1:1000 dilution; Dako/Agilent Technologies, Mulgrave, Vic, Australia); or a polyclonal antibody to collagen I (ab34710; 1:1000 dilution; Abcam). The equivalent loading of protein between samples was confirmed using a monoclonal antibody to the house-keeping protein, GAPDH (ab8245; 1:1000 dilution; Abcam). In each case, appropriate anti-mouse or anti-rabbit HRP conjugated secondary antibodies (Cell Signaling Technologies) were used, while blots were detected using the Clarity Western ECL substrate detection kit and quantified by densitometry with a ChemiDoc*™* MP Imaging System and Image Lab version 6.0 software (both from Bio-Rad Laboratories, Richmond, CA, United States). The density of each parameter was corrected for GAPDH protein levels, and then expressed relative to the indicated control group in each case, which was expressed as 1 in each case.

### Measurement of Caspase-1 and IL-1β Activity

As caspase-1 and IL-1β activity are difficult to measure at the cellular level (based on cell number) (Cáceres et al., 2019), BJ3 HDMFs were seeded at a density of 1–1.5x10^6^ cells/well in 12-well plates. HDMFs were then transfected with CTL or caspase-1 siRNA for 24 h. Transfected HDMFs were then treated either with T+L+A or T+L+A+R for 8 h. After 8 h, the media from nine sets of duplicate samples (18 ml) for each treatment group were pooled and concentrated down to 300 µl (x60) using Amicon^®^ Ultra-4 centrifugal filter units with a 10 kDa cut-off (Merck Millipore, Vic, Australia). Concentrated media samples were then assayed in triplicate using a R&D Systems Human IL-1β/IL-1F2 Quantikine ELISA and Human Caspase-1/ICE Quantikine ELISA Kits (In Vitro Technologies, Vic, Australia), according to manufacturer’s instructions.

### Statistical Analysis

All statistical analysis was performed using GraphPad Prism v7.0 (GraphPad Software Inc., CA, United States). All data was analysed *via* a one-way ANOVA with Neuman-Keuls post-hoc test for multiple comparisons between groups. In each case, results were expressed as the relative mean ± SEM with a p-value *<*0.05 considered statistically significant.

## Results

### Effects of RLX on NLRP3 Inflammasome Priming and Activation in Wild-Type HDMFs After 8 h

**Figure 1:**
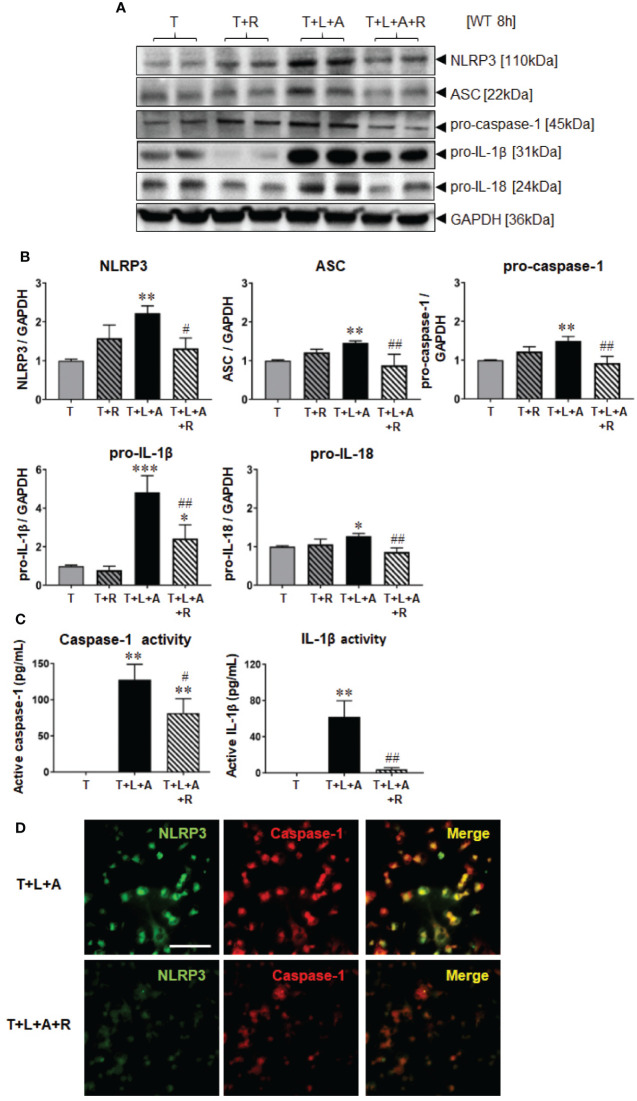
The effects of RLX on NLRP3 inflammasome priming and activity in T- and T+L+A-stimulated WT BJ3 HDMFs after 8 h. **(A)** Representative Western blots of NLRP3, ASC, pro-caspase-1, pro-IL-1β, and pro-IL-18 protein expression from HDMFs stimulated with TGF-β1 (T; 5 ng/ml), T+R (RLX; 100 ng/ml), T+L (LPS; 100 ng/ml) +A (ATP; 5 mM), or T+L+A+R over 8 h. **(B)** Also shown are the mean ± SEM of each end-point measured from each treatment group studied, corrected for GAPDH loading and expressed relative to the value in the T-treated group, which was expressed as 1 in each case; from n=6–8 separate experiments conducted in duplicate. **(C)** Additionally shown are the mean ± SEM levels of mature, secreted active caspase-1 (pg/ml) and active IL-1β (pg/ml) activity detected from the concentrated media of T-, T+L+A- and T+L+A+R-treated HDMFs after 8 h. *p<0.05, **p<0.01, ***p<0.001 vs. T-alone treated group; ^#^p<0.05, ^##^p<0.01 vs. T+L+A-treated group. **(D)** Representative images of NLRP3, caspase-1 and co-localization of NLRP3 and caspase-1 in T+L+A- vs. T+L+A+R-treated HDMFs confirm the presence of the NLRP3 inflammasome in the HDMFs studied, and the ability of RLX to inhibit the individual and co-localized components of the inflammasome. Scale bar; 100 μm.

Wild-type (WT) BJ3 HDFs were stimulated with LPS+ATP (L+A) or with TGF-β1 (T) to promote their differentiation into myofibroblasts (HDMFs) as well as T+L+A to promote the induction of the NLRP3 inflammasome in T-stimulated HDMFs for 8 h [the optimal time-point at which NLRP3 inflammasome activity was increased in myofibroblasts (Boza et al., 2016)]. While L+A-stimulated HDFs did not undergo any marked changes in NLRP3 inflammasome components (NLRP3, ASC, pro-caspase-1; **Supplementary Table 1**); T+L+A-stimulated HDMFs had significantly increased protein expression of measures of NLRP3 inflammasome priming, NLRP3, ASC and pro-caspase-1 (by 45–120%), as well as activity, pro-IL-1β (by ~4-fold) and pro-IL (by ~28%) after 8 h, as determined by Western blotting (**Figures 1A, B**, all p<0.05 vs. T alone). The T+L+A-stimulated increase in cellular pro-caspase-1 and pro-IL-1β protein expression correlated with a significant increase in maturely secreted caspase-1 and IL-1β activity (**Figure 1C**, both p<0.01 vs. T alone), as determined by ELISA analysis of concentrated media samples. RLX (R, 100 ng/ml) administration to T alone-stimulated HDMFs did not significantly affect any of these end-points measured. However, RLX administration to T+L+A-stimulated HDMFs significantly inhibited NLRP3, ASC, pro-caspase-1, and pro-IL-18 protein expression as well as mature IL-1β activity to levels that were no longer different to that measured in T alone-stimulated cells (**Figures 1B, C**, all p<0.05 vs. T+L+A); and partially inhibited pro-IL-1β protein expression (by ~63%) and mature caspase-1 levels (by ~36%) (**Figures 1A, C**, both p<0.05 vs. T+L+A; p<0.05 vs. T alone). RLX administration to T+L+A-stimulated WT HDMFs also reduced the degree of co-localization between NLRP3 and caspase-1 in these cells, compared to T+L+A-stimulated cells alone (**Figure 1D**).

### Effects of RLX on NLRP3 Inflammasome Priming/Activation and Fibrosis in WT HDMFs After 72 h

**Figure 2:**
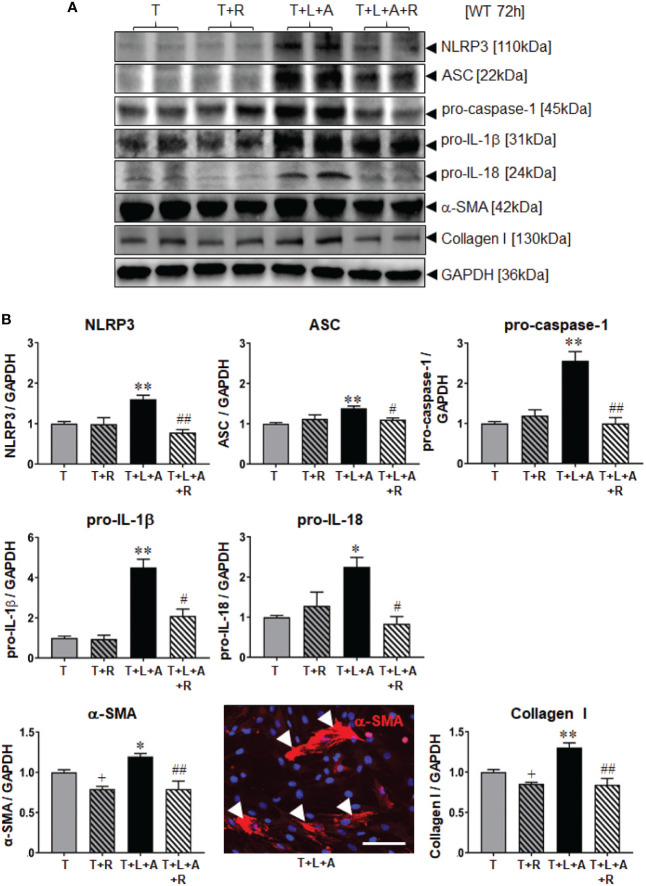
The effects of RLX on NLRP3 inflammasome priming and activity, and measures of fibrosis in T- and T+L+A-stimulated WT BJ3 HDMFs after 72 h. **(A)** Representative Western blots of NLRP3, ASC, pro-caspase-1, pro-IL-1β and pro-IL-18, α-SMA (used as a marker of myofibroblast differentiation), and collagen I protein expression from HDMFs stimulated with TGF-β1 (T; 5 ng/ml), T+R (RLX; 100 ng/ml), T+L(LPS; 100 ng/ml)+A(ATP; 5 mM), or T+L+A+R over 72 h. **(B)** Also shown are the mean ± SEM of each end-point measured from each treatment group studied, corrected for GAPDH loading and expressed relative to the value in the T-treated group, which was expressed as 1 in each case; from n=6–8 separate experiments conducted in duplicate. *p<0.05, **p<0.01 vs. T-alone treated group; ^#^p<0.05, ^##^p<0.01 vs. T+L+A-treated group. Immunofluoresence (red) staining of α-SMA in stress fibres (indicated by white arrows) in T+L+A-stimulated HDMFs confirmed that these stimulated cells were myofibroblasts after 72 h of culture. 4′,6-diamidino-2-phenylindole (DAPI; blue) staining was used to detect cell nuclei. Scale bar; 100 μm.

WT BJ3 HDFs were separately stimulated with L+A, T or T+L+A for 72 h (representing the time point at which changes in markers of fibrosis could be measured from myofibroblasts), and T or T+L+A-stimulated HDMFs co-treated with RLX (R) over the same time-period. Once again, L+A-stimulation of HDF failed to induce any marked changes in NLRP3 inflammasome components (NLRP3, ASC, pro-caspase-1; **Supplementary Table 1**). On the other hand, T+L+A-stimulated HDMFs had significantly increased protein expression of NLRP3, ASC, pro-caspase-1 (by ~38-155%), pro-IL-1β (by ~3.5-fold), and pro-IL-18 (by ~125%), which corresponded to a modest but significant increase in α-SMA (by ~20%) and collagen I (by ~30%) protein expression (**Figure 2**, all p<0.05 vs. T alone) after 72 h. RLX significantly inhibited α-SMA and collagen I protein expression in T alone-stimulated cells (**Figure 2**, by ~15-21%; both p<0.05 vs. T alone) in the absence of having any effects on measures of the NLRP3 inflammasome. However, RLX fully inhibited the T+L+A-stimulated increase in NLRP3, ASC and pro-caspase-1 (**Figure 2**, all p<0.05 vs. T+L+A); partially inhibited the T+L+A-stimulated increase in pro-IL-1β (**Figure 2**, by ~70%; p<0.01 vs. T+L+A; p<0.05 vs. T alone); and further inhibited the T+L+A-stimulated increase in levels of α-SMA and collagen I protein expression (**Figure 2**, both p<0.01 vs. T+L+A) to those that were measured from RLX treatment of T alone-stimulated cells (**Figure 2**).

### Validation of Lipofectamine-siRNA-Mediated Knock-Down of NLRP3 Inflammasome Components in WT HDMFs

**Figure 3:**
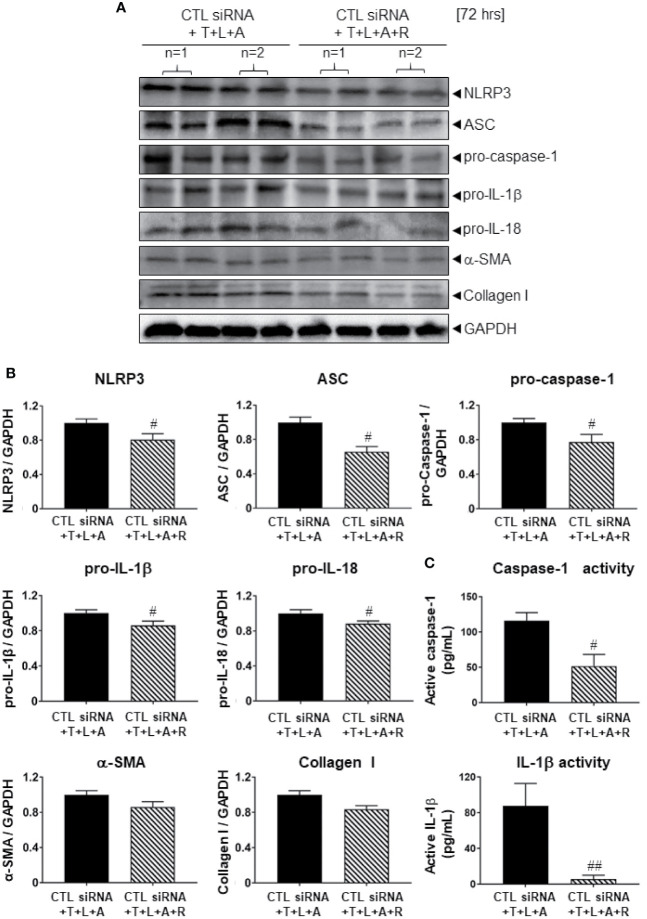
The effects of RLX on Lipofectamine-induced CTL siRNA transfection in NLRP3 inflammasome activated BJ3 HDMFs after 72 h. **(A)** Representative Western blots of NLRP3, ASC, pro-caspase-1, pro-IL-1β, pro-IL-18, α-SMA, and collagen-I protein expression in HDMFs treated with CTL siRNA ± T+L+A ± R treatment after 72 h. **(B)** Also shown are the mean ± SEM of each end-point measured from each treatment group studied, corrected for GAPDH loading and expressed relative to the value in the CTL siRNA+T+L+A-treated HDMF group, which was expressed as 1 in each case; from n=4–6 separate experiments conducted in duplicate. ^#^p<0.05 ^##^p<0.01 vs. CTL siRNA+T+L+A-treated group. **(C)** Additionally shown are the mean ± SEM levels of active caspase-1 (pg/ml) (*top panel*) and active IL-1β (pg/ml) (*bottom panel*) in CTL siRNA transfected HDFs treated with T+L+A or T+L+A+R after 72 h.

We first determined whether the control (CTL) siRNA obtained had any effects on the NLRP3 inflammasome-inhibitory and anti-fibrotic effects of RLX in HDMFs. Consistent with the data obtained from RLX-treated WT HDMFs after 72 h (**Figure 2**), RLX treatment of CTL siRNA transfected-HDMFs stimulated with T+L+A (CTL siRNA+T+L+A+R), significantly inhibited protein expression levels of NLRP3, ASC, pro-caspase-1, pro-IL-1β, pro-IL-18, α-SMA and collagen I (**Figures 3A, B**, all p<0.05 vs. CTL siRNA+T+L+A); as well as mature levels of caspase-1 and IL-1β activity (**Figure 3C**, both p<0.05 vs. CTL siRNA+T+L+A) after 72 h. These findings indicated that the anti-fibrotic effects of RLX in T+L+A-stimulated HDMFs were not altered by the presence of the CTL siRNA.

**Figure 4:**
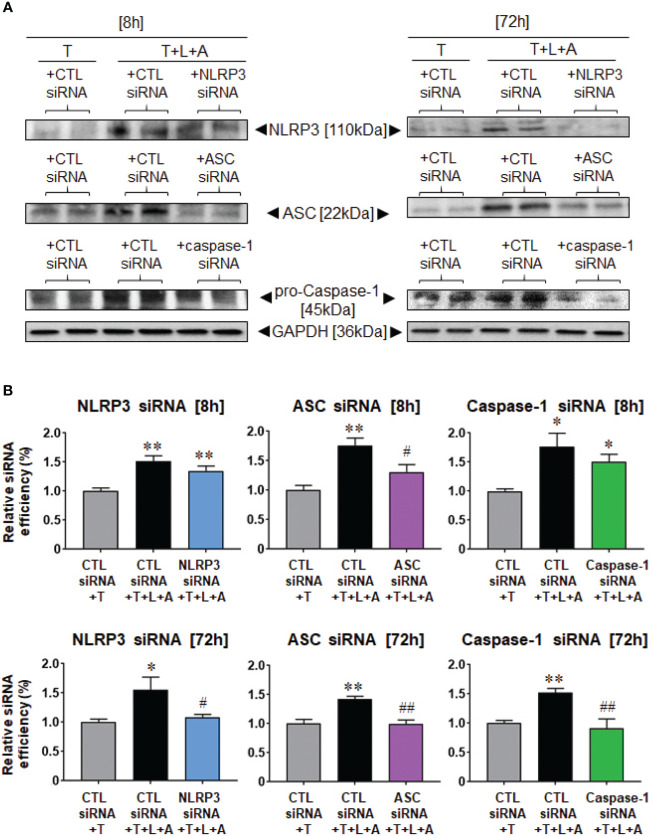
Lipofectamine-mediated siRNA-induced knock-down of NLRP3, ASC or pro-caspase-1 after 8 or 72 h in BJ3 HDMFs. **(A)** Representative Western blots of NLRP3, ASC and pro-caspase-1 protein expression in HDMFs treated with CTL siRNA+T, CTL siRNA+T+L+A, or NLRP3 siRNA+T+L+A, ASC siRNA+T+L+A, or caspase-1 siRNA+T+L+A, respectively, after 8 or 72 h (where siRNA were added for only 24 h for the 72 h end-points measured). **(B)** Also shown are the mean ± SEM of the transfection efficiency of each siRNA used against its target protein, at each time-point studied. Each measure was corrected for GAPDH loading and expressed relative to the value in the CTL siRNA+T-treated group, which was expressed as 1 in each case; from n=4–6 separate experiments conducted in duplicate. *p<0.05, **p<0.01 vs. CTL siRNA+T-treated group; ^#^p<0.05, ^##^p<0.01 vs. CTL siRNA+T+L+A-treated group.

We then examined the siRNA-induced knock-down efficiency of NLRP3, ASC or caspase-1 in T- and T+L+A-stimulated HDMFs after 8 h and 72 h, in comparison to the CTL siRNA, which should not have induced any knock-down of the end-points measured (**Figure 4**). Compared to CTL siRNA transfected-HDMFs that were stimulated with T alone, CTL siRNA transfected-HDMFs stimulated with T+L+A expressed significantly increased NLRP3, ASC, and pro-caspase-1 after 8 h (by ˜50%–75%) and 72 h (by ˜40%–55%) (**Figure 4**, all p<0.05 vs. CTL siRNA+T alone). siRNA-induced knock-down of NLRP3 (NLRP3 siRNA+T+L+A) or caspase-1 (caspase-1 siRNA+T+L+A) did not reduce NLRP3 or caspase-1 expression, respectively after 8 h, but induced complete knock-down of NLRP3 or caspase-1 expression, respectively after 72 h (**Figure 4**, both p<0.05 vs. CTL siRNA+T+L+A; no difference to CTL siRNA+T alone). In contrast, siRNA-induced knock-down of ASC (ASC siRNA+T+L+A) significantly reduced ASC expression from T+L+A-stimulated HDMFs at 8 h and to a greater extent at 72 h (**Figure 4**, both p<0.05 vs. CTL siRNA+T+L+A; difference to CTL siRNA+T alone). These findings confirmed the complete siRNA-induced knock-down of NLRP3, ASC, or caspase-1 from T+L+A-stimulated HDMFs after 72 h.

### The Effects of RLX on the Myofibroblast NLRP3 Inflammasome in the Absence or Presence of siRNA Treatment

**Figure 5:**
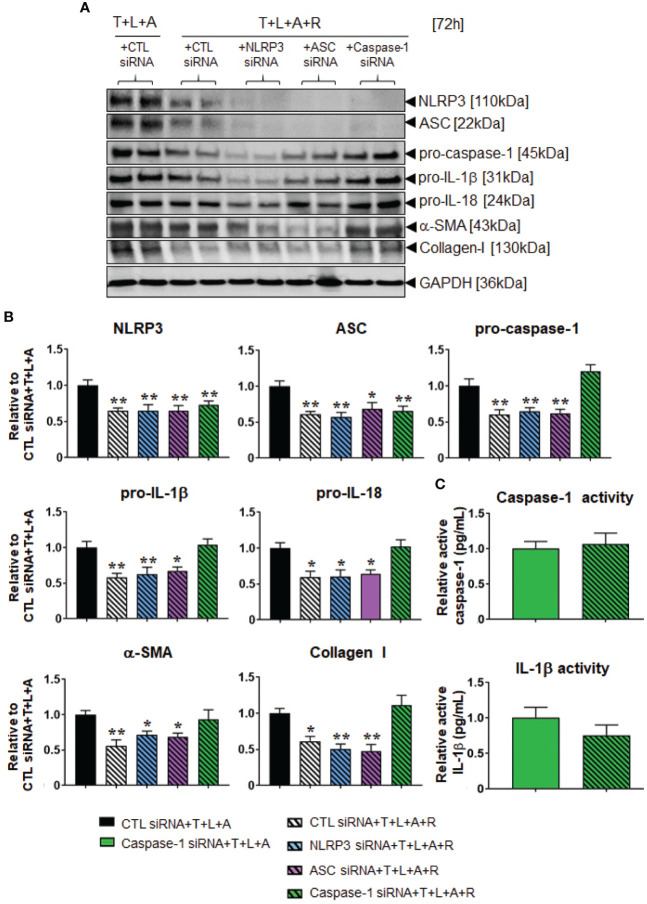
The effects of RLX in siRNA-transfected and NLRP3 inflammasome activated BJ3 HDMFs after 72 h. **(A)** Representative Western blots of NLRP3, ASC, pro-caspase-1, pro-IL-1β, pro-IL-18, α-SMA, and collagen I protein expression in HDMFs transfected with CTL siRNA for 24 h and subsequently stimulated with T+L+A ± R for 72 h; or NLRP3 siRNA, ASC siRNA, or caspase-1 siRNA for 24 h and subsequently stimulated with T+L+A+R for 72 h. **(B)** Also shown are the mean ± SEM of protein expression levels of each end-point measured, corrected for GAPDH loading and expressed relative to the value in the CTL siRNA+T+L+A-treated group; which was expressed as 1 in each case; from n=4–6 separate experiments conducted in duplicate. *p<0.05, **p<0.01 vs. the CTL siRNA+T+L+A-treated group. **(C)** Additionally shown are the relative mean ± SEM levels of active caspase-1 (pg/ml) (*top panel*) and active IL-1β (pg/ml) (*bottom panel*) in caspase-1 siRNA transfected HDFs treated with T+L+A or T+L+A+R after 72 h.

Following establishment of siRNA-mediated knock-down of NLRP3, ASC or caspase-1 in HDMFs, we next examined whether RLX modulated the myofibroblast NLRP3 inflammasome to mediate its anti-fibrotic actions. The stimulation of HDFs with T+L+A in the presence of CTL siRNA (black bars), NLRP3 siRNA (blue bars), ASC siRNA (purple bars), and caspase-1 siRNA (green bars), had comparable effects on measures of NLRP3 priming and activity as well as fibrosis (**Figures 5A, B****)**. In contrast, the administration of RLX to CTL siRNA transfected and T+L+A-stimulated HDMFs significantly downregulated measures of NLRP3 priming and activity, and fibrosis (**Figures 5A, B****)**, which was consistent with the effects of RLX shown in WT HDMFs (**Figures 2A, B**; in the absence of siRNA). These inhibitory effects of RLX were unaffected by the presence of siRNA targeting NLRP3 or ASC (**Figures 5A, B****)**. These findings suggested that RLX did not modulate NLRP3 or ASC within the myofibroblast NLRP3 inflammasome to inhibit pro-IL-1β and pro-IL-18. However, only in the presence of siRNA targeting caspase-1 (where caspase-1 was knocked-down prior to stimulation with T+L+A ± R) was the ability of RLX to downregulate caspase-1 (but not NLRP3 or ASC), pro-IL-1β, pro-IL-18, α-SMA, and collagen-I, completely abrogated, indicating that RLX was able to modulate caspase-1 within the myofibroblast NLRP3 inflammasome to mediate its anti-fibrotic effects (**Figures 5A, B****)**. To further consolidate these results, we measured the levels of mature caspase and IL-1β activity in caspase-1 siRNA transfected HDMFs treated with T+L+A and also separately with T+L+A+R (**Figure 5C**). In the presence of siRNA targeting caspase-1, the ability of RLX to downregulate caspase-1 and IL-1β activity was suppressed. These findings confirmed that RLX could modulate the myofibroblast NLRP3 inflammasome at the level of caspase-1 to inhibit pro-IL-1β, pro-IL-18, myofibroblast differentiation, and myofibroblast-induced collagen I deposition.

## Discussion

RLX has emerged as a rapidly-acting anti-fibrotic therapy, and at least at the experimental level, as a more efficacious anti-fibrotic compared to the ACE inhibitor, enalapril (Samuel et al., 2014). It was previously shown that RLX mediated its anti-fibrotic effects through direct (Kocan et al., 2017) or indirect (Mookerjee et al., 2009; Wang et al., 2016) activation of cGMP, the latter involving nNOS-NO-sGC-cGMP signaling, to inhibit TGF-β1 signal transduction at the level of intracellular Smad2 (Mookerjee et al., 2009; Samuel et al., 2014; Wang et al., 2016; Kocan et al., 2017) and/or Smad3 (Kocan et al., 2017) in myofibroblasts. This consistently resulted in the RLX-induced inhibition of the pro-fibrotic influence of TGF-β1 on myofibroblast differentiation and myofibroblast-induced collagen deposition, in the absence of RLX having any direct effects on collagen (Unemori et al., 1996; Samuel et al., 2004). Given that the inflammatory cytokine, IL-1β, can promote fibrosis progression through an autocrine loop with the TGF-β1/Smad3 axis (Bonniaud et al., 2005; Aoki et al., 2006), and that RLX was found to inhibit collagen synthesis from either IL-1β or TGF-β1-stimulated HDMFs (Unemori and Amento, 1990), our recent attention turned to how RLX disrupted the TGF-β1/IL-1β interaction. As RLX was also found to inhibit renal fibrosis through a TLR-4-dependent mechanism (Chen et al., 2017), which is a known inducer of the myofibroblast NLRP3 inflammasome (Boza et al., 2016), and IL-1β along with IL-18 can be produced by this inflammasome (Artlett and Thacker, 2015), we investigated whether RLX targeted TGF-β1 and TLR-4-induced NLRP3 inflammasome activity in myofibroblasts to mediate its anti-fibrotic actions.

**Figure 6:**
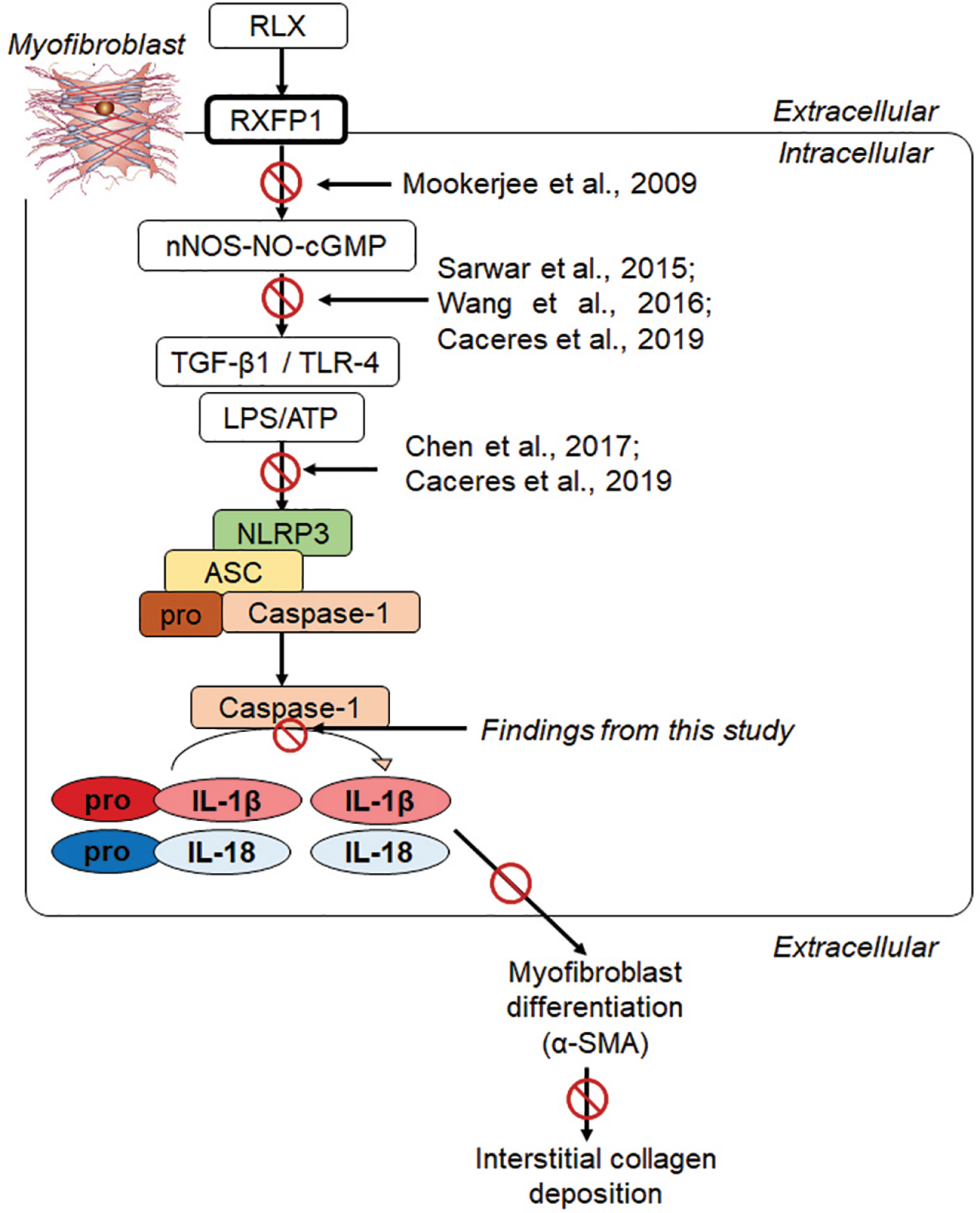
Progressive studies that we and others have published, have led to our recent findings showing that RLX signaled through a RXFP1-nNOS-TLR-4-dependent mechanism in human cardiac myofibroblasts to inhibit measures of myofibroblast NLRP3 inflammasome priming and activity, as well as myofibroblast differentiation and myofibroblast-induced collagen I deposition (Cáceres et al., 2019). We now show in this study that RLX can additionally modulate the myofibroblast NLRP3 inflammasome at the level of caspase-1 to mediate its anti-fibrotic actions; as the ability of RLX to inhibit IL-1β, pro-IL-18, myofibroblast differentiation and collagen I deposition was specifically abrogated by siRNA-induced knock-down of caspase-1, but not of NLRP3 or ASC. Our collective findings suggest that RLX can target the TGF-β1-TLR-4 axis (upstream of the NLRP3 inflammasome) to inhibit NLRP3, ASC and pro-caspase-1; and also target caspase-1 activity (within the NLRP3 inflammasome) to inhibit the pro-fibrotic contributions of IL-1β and IL-18 that is produced by the NLRP3 inflammasome, on myofibroblast differentiation (α-SMA expression) and collagen I deposition.

Our recent findings revealed that RLX signaled through a RXFP1-nNOS-TLR-4-dependent mechanism in human cardiac myofibroblasts *in vitro* and in a murine model of isoproterenol-induced cardiomyopathy *in vivo* to inhibit measures of myofibroblast NLRP3 inflammasome priming and activity, as well as myofibroblast differentiation and collagen I deposition (Cáceres et al., 2019). These findings extended the above-mentioned studies in demonstrating that RLX could ameliorate NLRP3 inflammasome activity in myofibroblasts to inhibit the pro-fibrotic interaction between TGF-β1, TLR-4 and IL-1β/IL-18 (**Figure 6**) on myofibroblast differentiation and myofibroblast-induced interstitial collagen deposition. Furthermore, they somewhat confirmed previous reports which showed that RLX could inhibit the pro-inflammatory and pro-fibrotic effects of IL-1β and IL-18 *in vivo*, but which did not investigate NLRP3 inflammasome activity in the experimental models studied. Despite these novel findings, it remained unclear as to whether RLX modulated the myofibroblast NLRP3 inflammasome to mediate its anti-fibrotic effects or was only able to indirectly regulating the inflammasome at the level of the TGF-β1-TLR-4 axis on cardiac myofibroblasts (Boza et al., 2016). The findings from this study, now show for the first time, that RLX can directly modulate the myofibroblast NLRP3 inflammasome at the level of caspase-1 to mediate its anti-fibrotic actions, as the ability of RLX to inhibit pro-IL-1β, pro-IL-18, myofibroblast differentiation and collagen I deposition was specifically abrogated by siRNA-induced knock-down of caspase-1, but not of NLRP3 or ASC. Collectively, our findings suggest firstly that RLX (acting through RXFP1) can target the TGF-β1-TLR-4 axis (upstream of the NLRP3 inflammasome) to inhibit NLRP3 and ASC; and secondly, through disruption of the TGF-β1-TLR-4 axis and pro-caspase 1 (within the inflammasome), RLX can down-regulate caspase-1 activity to dampen the pro-fibrotic contributions of IL-1β and IL-18, that is produced by the myofibroblast NLRP3 inflammasome, on myofibroblast differentiation and collagen-I deposition (**Figure 6**). Our added findings that RLX could disrupt TGF-β1-induced myofibroblast differentiation and collagen-I deposition, in the absence of L+A-induced NLRP3 inflammasome activity, additionally suggests that RLX does not exclusively regulate the myofibroblast NLRP3 inflammasome to mediate its anti-fibrotic actions. Consistent with this, studies have shown that RLX can also suppress the upregulation of several chemokines and cytokines independently of IL-1β and/or IL-18 regulation (reviewed in Bathgate et al., 2013; Samuel et al., 2017). Our findings show that RLX is able to disrupt the TGF-β1-IL-1β and TGF-β1-IL-18 axes through the NLRP3 inflammasome on myofibroblasts.

Furthermore, our findings shown in **Supplementary Table 1** suggest that pro-caspase-1 and caspase-1 activity can be regulated independently of NLRP3 and/or ASC. As T-alone (without L+A) was found to stimulate pro-caspase-1 protein expression after 72 h (**Supplementary Table 1**), in the absence of NLRP3 or ASC in HDMFs, this provides evidence to suggest that pro-caspase-1 can be regulated independently of NLRP3 and ASC, and independently of NLRP3 inflammasome induction. This notion is in line with previous studies which demonstrated that caspase-1 activity can be regulated upstream of the NLRP3 inflammasome, at the level of TLR-4 and NF-κB activation (Kahlenberg et al., 2005; Franchi et al., 2009). On the other hand, as T+L+A stimulation promoted NLRP3-dependent caspase-1 and IL-1β activity in HDMFs, this confirmed the NLRP3-dependent induction of caspase-1 and IL-1β activity, consistent with previous studies (Martinon et al., 2002; Sutterwala et al., 2006; Bauernfeind et al., 2009; Cáceres et al., 2019). Therefore, it is possible that caspase-1 can be regulated *via* NLRP3-dependent and -independent mechanisms. Hence, further work is required to determine how RLX specifically targets caspase-1 activity on myofibroblasts.

Nevertheless, our findings overlap but are distinct from a previous study, which demonstrated that RLX could attenuate caspase-1 activity in an *in vivo* model of ischemia/reperfusion injury *via* an eNOS-dependent mechanism (Valle Raleigh et al., 2017), in which the direct involvement of the NLRP3 inflammasome was not provided (Valle Raleigh et al., 2017). As NO has been found to inhibit caspase-1, IL-1β, and IL-18 release from macrophages (Collino et al., 2013), it was suggested that the ability of RLX to promote eNOS-induced NO correlated with the RLX-induced attenuation of NLRP3 inflammasome-induced caspase-1 that was measured in the murine model studied (Valle Raleigh et al., 2017). However, several lines of evidence have indicated RLX to signal *via* a nNOS-dependent mechanism in myofibroblasts. Rat renal myofibroblasts (Mookerjee et al., 2009; Chow et al., 2012) and human cardiac myofibroblasts (Sarwar et al., 2015) express nNOS and iNOS but lack eNOS, with RLX shown to specifically up-regulate nNOS expression in these myofibroblasts (Mookerjee et al., 2009; Chow et al., 2012; Sarwar et al., 2015). Furthermore, pharmacological blockade of nNOS activity in rat renal myofibroblasts has been shown to inhibit the anti-fibrotic effects of RLX on TGF-β1 signal transduction and myofibroblast differentiation (Mookerjee et al., 2009). Therefore, it would appear that RLX signals through a nNOS-dependent mechanism in myofibroblasts to inhibit TLR-4-NLRP3 inflammasome-caspase-1-IL-1β/IL-18 activity. On the other hand, RLX may regulate the NLRP3 inflammasome in other cells such as macrophages through an eNOS-NO- (Sogawa et al., 2018) or iNOS-NO- (Mao et al., 2013) dependent mechanism. Irrespective of how NO is produced by various cells, both our findings and those of Valle Raleigh (Valle Raleigh et al., 2017) and colleagues suggest that the RLX-induced promotion of NO can target caspase-1 release and activity within the NLRP3 inflammasome in multiple cell types, and its subsequent ability to activate IL-1β and IL-18.

The expression and activity of the NLRP3 inflammasome from different cell types (including various innate immune cells and myofibroblasts) is likely to be varied during wound healing-induced inflammation, tissue remodeling and eventually fibrogenesis (Yin et al., 2009). During the inflammatory phase of the wound healing response, the macrophage NLRP3 inflammasome is highly expressed and plays a fundamental role in secreting IL-1β and IL-18, which along with the TGF-β1 that is secreted by macrophages, contribute to the activation of resident ECM-producing fibroblasts (Wynn and Barron, 2010). Although not expressed as highly as their macrophage counterparts, myofibroblast NLRP3 inflammasome components, such as NLRP3 and ASC, have been detected during the early stages of wound healing in response to injury (Mezzaroma et al., 2011; Gan et al., 2018). However, the IL-1β that is secreted by the myofibroblast NLRP3 inflammasome has been demonstrated to promote the effects of TGF-β1 on human fibroblast proliferation and ECM synthesis, eventually contributing to adverse tissue remodeling and fibrosis (Vesey et al., 2002). Similarly, the epithelial cell NLRP3 inflammasome has been found to contribute to epithelial-mesenchymal-transition (EMT) during wound healing-associated inflammation in response to tissue injury, which over time, contributes to fibrogenesis *via* an interaction with TGF-β1 (Liu, 2004; Wang et al., 2013). Hence, further work is required to determine whether RLX universally targets NLRP3 inflammasome activity, and indeed the activity of other inflammasomes that contribute to fibrosis progression, or has temporal effects in suppressing over-activated inflammasome activity in a cell-dependent manner, to mediate its anti-fibrotic effects.

The findings from this study have provided further insight into the molecular mechanisms by which RLX mediates its anti-fibrotic actions on TGF-β1 signal transduction and its interaction with the myofibroblast NLRP3 inflammasome. It is now clear that RLX can act at multiple levels to disrupt the pro-fibrotic influence of TGF-β1, through its ability to promote nNOS-induced NO and cGMP in myofibroblasts. RLX can disrupt TGF-β1 signal transduction at the level of Smad2 and/or Smad3 (Heeg et al., 2005; Mookerjee et al., 2009; Sassoli et al., 2013; Samuel et al., 2014; Wang et al., 2016; Kocan et al., 2017). It can also ameliorate the interaction between TGF-β1 and TLR-4 (up-stream of the NLRP3 inflammasome) (Cáceres et al., 2019) and caspase-1 activity (at the level of the NLRP3 inflammasome) to suppress IL-1β and IL-18 activity, as well as the interaction between TGF-β1 and secreted IL-1β (down-stream of the NLRP3 inflammasome) to mediate its anti-fibrotic actions (**Figure 6**). In summary, these findings highlight the therapeutic relevance of targeting the myofibroblast NLRP3 inflammasome as a means of treating the fibrosis that is a key cause of tissue dysfunction and failure. In this regard, natural (Molteni et al., 2018) and pharmacological inhibitors (Matsunaga et al., 2011) of TLR-4, NLRP3 (Coll et al., 2015), caspase-1 (Kudelova et al., 2015), or IL-1β (Kudelova et al., 2015; Emmi et al., 2018) activity may limit the contribution of the NLRP3 inflammasome to fibrosis progression, while avoiding the redundancy and side-effects associated with TGF-β1 blockade alone. However, future studies aimed at combining these drugs with RLX may lead to more efficacious blockade of fibrosis through suppression of the TGF-β1-TLR-4-NLRP3 inflammasome axis.

## Data Availability Statement

All datasets generated for this study are included in the article/**Supplementary Material**.

## Author Contributions

AP and CS conceived, designed and supervised the study. AP, AY, and TG performed all the experiments. AP, AY, TG, and CS analyzed the data. AP, AY, TG, and CS contributed to writing of the manuscript.

## Funding

This research was supported in part by a Monash Biomedicine Discovery Institute Fellowship to CS.

## Conflict of Interest

The authors declare that the research was conducted in the absence of any commercial or financial relationships that could be construed as a potential conflict of interest.

## References

[B1] Aoki H. Ohnishi H. Hama K. Ishijima T. Satoh Y. Hanatsuka K. (2006). Autocrine loop between TGF-beta1 and IL-1beta through Smad3- and ERK-dependent pathways in rat pancreatic stellate cells. Am. J. Physiol. Cell. Physiol. 290, C1100–C1108. 10.1152/ajpcell.00465.2005 16371439

[B2] Artlett C. M. Thacker J. D. (2015). Molecular activation of the NLRP3 Inflammasome in fibrosis: common threads linking divergent fibrogenic diseases. Antioxid. Redox Signal. 22, 1162–1175. 10.1089/ars.2014.6148 25329971

[B3] Bathgate R. A. Halls M. L. Van Der Westhuizen E. T. Callander G. E. Kocan M. Summers R. J. (2013). Relaxin family peptides and their receptors. Physiol. Rev. 93, 405–480. 10.1152/physrev.00001.2012 23303914

[B4] Bauernfeind F. G. Horvath G. Stutz A. Alnemri E. S. MacDonald K. Speert D. (2009). Cutting edge: NF-κB activating pattern recognition and cytokine receptors license NLRP3 inflammasome activation by regulating NLRP3 expression. J. Immunol. 183, 787–791. 10.4049/jimmunol.0901363 19570822 PMC2824855

[B5] Beiert T. Tiyerili V. Knappe V. Effelsberg V. Linhart M. Stockigt F. (2017). Relaxin reduces susceptibility to post-infarct atrial fibrillation in mice due to anti-fibrotic and anti-inflammatory properties. Biochem. Biophys. Res. Commun. 490, 643–649. 10.1016/j.bbrc.2017.06.091 28634079

[B6] Bonniaud P. Margetts P. J. Ask K. Flanders K. Gauldie J. Kolb M. (2005). TGF-beta and Smad3 signaling link inflammation to chronic fibrogenesis. J. Immunol. 175, 5390–5395. 10.4049/jimmunol.175.8.5390 16210645

[B7] Boza P. Ayala P. Vivar R. Humeres C. Caceres F. T. Munoz C. (2016). Expression and function of toll-like receptor 4 and inflammasomes in cardiac fibroblasts and myofibroblasts: IL-1beta synthesis, secretion, and degradation. Mol. Immunol. 74, 96–105. 10.1016/j.molimm.2016.05.001 27174187

[B8] Cáceres F. T. Gaspari T. A. Samuel C. S. Pinar A. A. (2019). Serelaxin inhibits the pro-fibrotic TGF-beta1/IL-1beta axis by targeting TLR-4 and the NLRP3 inflammasome on myofibroblasts. FASEB J. 33, 14717–14733. 10.1096/fj.201901079RR 31689135

[B9] Chen L. Sha M. L. Li D. Zhu Y. P. Wang X. J. Jiang C. Y. (2017). Relaxin abrogates renal interstitial fibrosis by regulating macrophage polarization via inhibition of Toll-like receptor 4 signaling. Oncotarget 8, 21044–21053. 10.18632/oncotarget.15483 28416741 PMC5400564

[B10] Chow B. S. Chew E. G. Zhao C. Bathgate R. A. Hewitson T. D. Samuel C. S. (2012). Relaxin signals through a RXFP1-pERK-nNOS-NO-cGMP-dependent pathway to up-regulate matrix metalloproteinases: the additional involvement of iNOS. PloS One 7, e42714. 10.1371/journal.pone.0042714 22936987 PMC3425563

[B11] Coll R. C. Robertson A. A. Chae J. J. Higgins S. C. Munoz-Planillo R. Inserra M. C. (2015). A small-molecule inhibitor of the NLRP3 inflammasome for the treatment of inflammatory diseases. Nat. Med. 21, 248–255. 10.1038/nm.3806 25686105 PMC4392179

[B12] Collino M. Rogazzoi M. Pini A. Benetti E. Rosa A. C. Chiazza F. (2013). Acute treatment with relaxin protects the kidney against ischaemia/reperfusion injury. J. Cell. Mol. Med. 17, 1494–1505. 10.1111/jcmm.12120 24079335 PMC4117562

[B13] Du X. J. Bathgate R. A. Samuel C. S. Dart A. M. Summers R. J. (2010). Cardiovascular effects of relaxin: from basic science to clinical therapy. Nat. Rev. Cardiol. 7, 48–58. 10.1038/nrcardio.2009.198 19935741

[B14] Emmi G. Urban M. L. Imazio M. Gattorno M. Maestroni S. Lopalco G. (2018). Use of Interleukin-1 blockers in pericardial and cardiovascular diseases. Curr. Cardiol. Rep. 20, 61. 10.1007/s11886-018-1007-6 29904899

[B15] Franchi L. Eigenbrod T. Muñoz-Planillo R. Nuñez G. (2009). The inflammasome: a caspase-1-activation platform that regulates immune responses and disease pathogenesis. Nat. Immunol. 10, 241–247. 10.1038/ni.1703 19221555 PMC2820724

[B16] Frangogiannis N. G. (2019). Cardiac fibrosis: Cell biological mechanisms, molecular pathways and therapeutic opportunities. Mol. Aspects Med. 65, 70–99. 10.1016/j.mam.2018.07.001 30056242

[B17] Gan W. Ren J. Li T. Lv S. Li C. Liu Z. (2018). The SGK1 inhibitor EMD638683, prevents Angiotensin II-induced cardiac inflammation and fibrosis by blocking NLRP3 inflammasome activation. Biochim. Biophys. Acta Mol. Basis. Dis. 1864, 1–10. 10.1016/j.bbadis.2017.10.001 28986310

[B18] Gasse P. Mary C. Guenon I. Noulin N. Charron S. Schnyder-Candrian S. (2007). IL-1R1/MyD88 signaling and the inflammasome are essential in pulmonary inflammation and fibrosis in mice. J. Clin. Invest. 117, 3786–3799. 10.1172/JCI32285 17992263 PMC2066195

[B19] Heeg M. H. Koziolek M. J. Vasko R. Schaefer L. Sharma K. Muller G. A. (2005). The antifibrotic effects of relaxin in human renal fibroblasts are mediated in part by inhibition of the Smad2 pathway. Kidney Int. 68, 96–109. 10.1111/j.1523-1755.2005.00384.x 15954899

[B20] Hossain M. A. Rosengren K. J. Samuel C. S. Shabanpoor F. Chan L. J. Bathgate R. A. D. (2011). The minimal active structure of human relaxin-2. J. Biol. Chem. 286, 37555–37565. 10.1074/jbc.M111.282194 21878627 PMC3199501

[B21] Humeres C. Frangogiannis N. G. (2019). Fibroblasts in the infarcted, remodeling, and failing heart. JACC Basic Transl. Sci. 4, 449–467. 10.1016/j.jacbts.2019.02.006 31312768 PMC6610002

[B22] Kahlenberg J. M. Lundberg K. C. Kertesy S. B. Qu Y. Dubyak G. R. (2005). Potentiation of caspase-1 activation by the P2X7 receptor is dependent on TLR signals and requires NF-κB-driven protein synthesis. J. Immunol. 175, 7611–7622. 10.4049/jimmunol.175.11.7611 16301671

[B23] Kocan M. Sarwar M. Ang S. Y. Xiao J. Marugan J. J. Hossain M. A. (2017). ML290 is a biased allosteric agonist at the relaxin receptor RXFP1. Sci. Rep. 7, 2968–2982. 10.1038/s41598-017-02916-5 28592882 PMC5462828

[B24] Kolb M. Margetts P. J. Anthony D. C. Pitossi F. Gauldie J. (2001). Transient expression of IL-1beta induces acute lung injury and chronic repair leading to pulmonary fibrosis. J. Clin. Invest. 107, 1529–1536. 10.1172/JCI12568 11413160 PMC200196

[B25] Kudelova J. Fleischmannova J. Adamova E. Matalova E. (2015). Pharmacological caspase inhibitors: research towards therapeutic perspectives. J. Physiol. Pharmacol. 66, 473–482. 10.1038/nm.3806 26348072

[B26] Li L. Zhao Q. Kong W. (2018). Extracellular matrix remodeling and cardiac fibrosis. Matrix Biol. 68-69, 490–506. 10.1016/j.matbio.2018.01.013 29371055

[B27] Liu Y. (2004). Epithelial to mesenchymal transition in renal fibrogenesis: pathologic significance, molecular mechanism, and therapeutic intervention. J. Am. Soc Nephrol. 15, 1–12. 10.1097/01.asn.0000106015.29070.e7 14694152

[B28] Mangan M. S. J. Olhava E. J. Roush W. R. Seidel H. M. Glick G. D. Latz E. (2018). Targeting the NLRP3 inflammasome in inflammatory diseases. Nat. Rev. Drug Discovery 17, 588–606. 10.1038/nrd.2018.97 30026524

[B29] Mao K. Chen S. Chen M. Ma Y. Wang Y. Huang B. (2013). Nitric oxide suppresses NLRP3 inflammasome activation and protects against LPS-induced septic shock. Cell Res. 23, 201–212. 10.1371/journal.pone.0203823 23318584 PMC3567828

[B30] Martinon F. Burns K. Tschopp J. (2002). The inflammasome: a molecular platform triggering activation of inflammatory caspases and processing of proIL-1β. Mol. Cell. 10, 417–426. 10.1016/s1097-2765(02)00599-3 12191486

[B31] Matsunaga N. Tsuchimori N. Matsumoto T. Ii M. (2011). TAK-242 (resatorvid), a small-molecule inhibitor of toll-like receptor (TLR)-4 signaling, binds selectively to TLR-4 and interferes with interactions between TLR-4 and its adaptor molecules. Mol. Pharmacol. 79, 34–41. 10.1124/mol.110.068064 20881006

[B32] Mezzaroma E. Toldo S. Farkas D. Seropian I. M. Van Tassell B. W. Salloum F. N. (2011). The inflammasome promotes adverse cardiac remodeling following acute myocardial infarction in the mouse. Proc. Natl. Acad. Sci. U.S.A. 108, 19725–19730. 10.1073/pnas.1108586108 22106299 PMC3241791

[B33] Molteni M. Bosi A. Rossetti C. (2018). Natural products with toll-like receptor 4 antagonist activity. Int. J. Inflam. 2018, 2859135–2859144‬. 10.1155/2018/2859135‬ PMC5852877 29686833

[B34] Mookerjee I. Hewitson T. D. Halls M. L. Summers R. J. Mathai M. L. Bathgate R. A. (2009). Relaxin inhibits renal myofibroblast differentiation via RXFP1, the nitric oxide pathway, and Smad2. FASEB J. 23, 1219–1229. 10.1096/fj.08-120857 19073841

[B35] Nistri S. Bigazzi M. Bani D. (2007). Relaxin as a cardiovascular hormone: physiology, pathophysiology and therapeutic promises. Cardiovasc. Hematol. Agents Med. Chem. 5, 101–108. 10.2174/187152507780363179 17430134

[B36] Rodriguez-Alcazar J. F. Ataide M. A. Engels G. Schmitt-Mabmunyo C. Garbi N. Kastenmuller W. (2019). Charcot-Leyden crystals activate the NLRP3 inflammasome and cause IL-1beta inflammation in human macrophages. J. Immunol. 202, 550–558. 10.4049/jimmunol.1800107 30559319

[B37] Samuel C. S. Unemori E. N. Mookerjee I. Bathgate R. A. Layfield S. L. Mak J. (2004). Relaxin modulates cardiac fibroblast proliferation, differentiation and collagen production and reverses cardiac fibrosis in vivo. Endocrinology 145, 4125–4133. 10.1210/en.2004-0209 15155573

[B38] Samuel C. S. Bodaragama H. Chew J. Y. Widdop R. E. Royce S. G. Hewitson T. D. (2014). Serelaxin is a more efficacious antifibrotic than enalapril in an experimental model of heart disease. Hypertension 64, 315–322. 10.1161/HYPERTENSIONAHA.114.03594 24866131

[B39] Samuel C. S. Royce S. G. Hewitson T. D. Denton K. M. Cooney T. E. Bennett R. G. (2017). Anti-fibrotic actions of relaxin. Br. J. Pharmacol. 174, 962–976. 10.1111/bph.13529 27250825 PMC5406285

[B40] Sarwar M. Samuel C. S. Bathgate R. A. Stewart D. R. Summers R. J. (2015). Serelaxin-mediated signal transduction in human vascular cells: bell-shaped concentration-response curves reflect differential coupling to G proteins. Br. J. Pharmacol. 172, 1005–1019. 10.1111/bph.12964 25297987 PMC4314191

[B41] Sassoli C. Chellini F. Pini A. Tani A. Nistri S. Nosi D. (2013). Relaxin prevents cardiac fibroblast-myofibroblast transition via notch-1-mediated inhibition of TGF-beta/Smad3 signaling. PloS One 8, e63896–e63906. 10.1371/journal.pone.0063896 23704950 PMC3660557

[B42] Schroer A. K. Merryman W. D. (2015). Mechanobiology of myofibroblast adhesion in fibrotic cardiac disease. J. Cell Sci. 128, 1865–1875. 10.1242/jcs.162891 25918124 PMC4457157

[B43] Sogawa Y. Nagasu H. Itano S. Kidokoro K. Taniguchi S. Takahashi M. (2018). The eNOS-NO pathway attenuates kidney dysfunction via suppression of inflammasome activation in aldosterone-induced renal injury model mice. PloS One 13, e0203823–e0203840. 10.1371/journal.pone.0203823 30281670 PMC6169882

[B44] Sutterwala F. S. Ogura Y. Szczepanik M. Lara-Tejero M. Lichtenberger G. S. Grant E. P. (2006). Critical role for NALP3/CIAS1/Cryopyrin in innate and adaptive immunity through its regulation of caspase-1. Immunity 24, 317–327. 10.1016/j.immuni.2006.02.004 16546100

[B45] Unemori E. N. Amento E. P. (1990). Relaxin modulates synthesis and secretion of procollagenase and collagen by human dermal fibroblasts. J. Biol. Chem. 265, 10681–10685. 2162358

[B46] Unemori E. N. Pickford L. B. Salles A. L. Piercy C. E. Grove B. H. Erikson M. E. (1996). Relaxin induces an extracellular matrix-degrading phenotype in human lung fibroblasts in vitro and inhibits lung fibrosis in a murine model in vivo. J. Clin. Invest. 98, 2739–2745. 10.1172/JCI119099 8981919 PMC507738

[B47] Valle Raleigh J. Mauro A. G. Devarakonda T. Marchetti C. He J. Kim E. (2017). Reperfusion therapy with recombinant human relaxin-2 (Serelaxin) attenuates myocardial infarct size and NLRP3 inflammasome following ischemia/reperfusion injury via eNOS-dependent mechanism. Cardiovasc. Res. 113, 609–619. 10.1093/cvr/cvw246 28073832

[B48] Vesey D. A. Cheung C. Cuttle L. Endre Z. Gobe G. Johnson D. W. (2002). Interleukin-1beta stimulates human renal fibroblast proliferation and matrix protein production by means of a transforming growth factor-beta-dependent mechanism. J. Lab. Clin. Med. 140, 342–350. 10.1067/mlc.2002.128468 12434136

[B49] Wang W. Wang X. Chun J. Vilaysane A. Clark S. French G. (2013). Inflammasome-independent NLRP3 augments TGF-beta signaling in kidney epithelium. J. Immunol. 190, 1239–1249. 10.4049/jimmunol.1201959 23264657

[B50] Wang C. Kemp-Harper B. K. Kocan M. Ang S. Y. Hewitson T. D. Samuel C. S. (2016). The anti-fibrotic actions of relaxin are mediated through a NO-sGC-cGMP-dependent pathway in renal myofibroblasts *in vitro* and enhanced by the NO donor, diethylamine NONOate. Front. Pharmacol. 7, 91. 10.3389/fphar.2016.00091 27065874 PMC4815292

[B51] Wynn T. A. Barron L. (2010). Macrophages: master regulators of inflammation and fibrosis. Semin. Liver Dis. 30, 245–257. 10.1055/s-0030-1255354 20665377 PMC2924662

[B52] Wynn T. A. Ramalingam T. R. (2012). Mechanisms of fibrosis: therapeutic translation for fibrotic disease. Nat. Med. 18, 1028–1040. 10.1038/nm.2807 22772564 PMC3405917

[B53] Wynn T. A. (2008). Cellular and molecular mechanisms of fibrosis. J. Pathol. 214, 199–210. 10.1002/path.2277 18161745 PMC2693329

[B54] Yin Y. Yan Y. Jiang X. Mai J. Chen N. C. Wang H. (2009). Inflammasomes are differentially expressed in cardiovascular and other tissues. Int. J. Immunopathol. Pharmacol. 22, 311–322. 10.1177/039463200902200208 19505385 PMC2847797

[B55] Zhou W. Chen C. Chen Z. Liu L. Jiang J. Wu Z. (2018). NLRP3: a novel mediator in cardiovascular disease. J. Immunol. Res. 2018, 5702103–5702111. 10.1155/2018/5702103 29850631 PMC5911339

