# Supplementary figures and images for: Relaxin Can Mediate Its Anti-Fibrotic Effects by Targeting the Myofibroblast NLRP3 Inflammasome at the Level of Caspase-1
Source: Front Pharmacol. 2020 Aug 4;11:1201. doi: 10.3389/fphar.2020.01201 (PMC7417934; doi:10.3389/fphar.2020.01201)

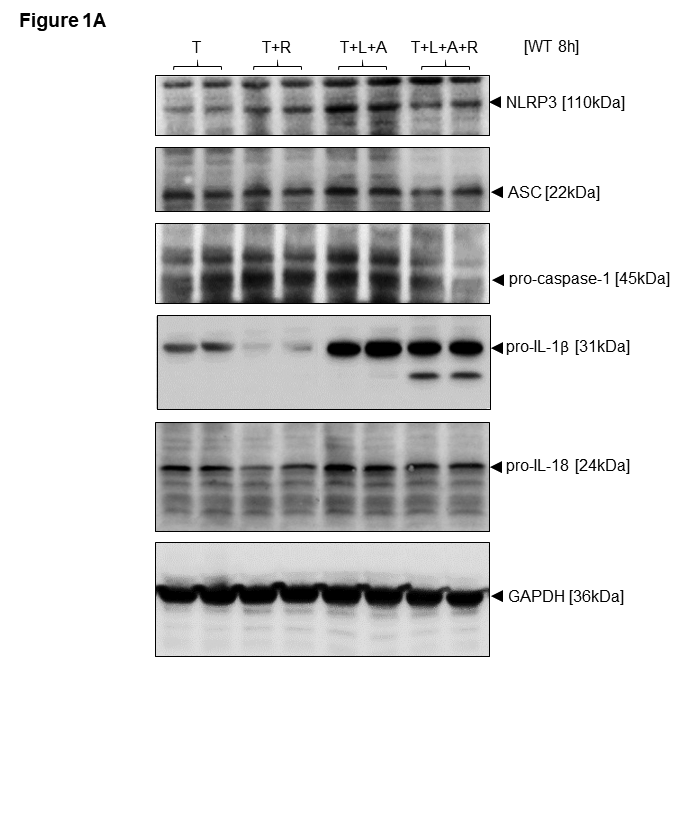

Supplement: Supplementary file 1 [file DataSheet_1.zip › Suppl_Figure 1A.tif]

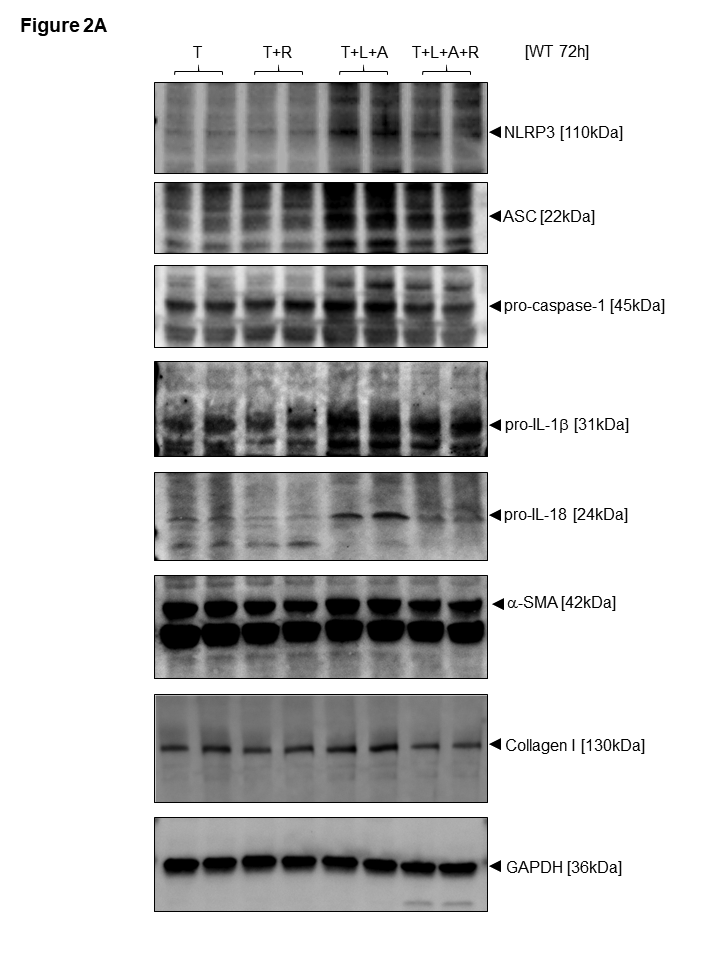

Supplement: Supplementary file 1 [file DataSheet_1.zip › Suppl_Figure 2A.tif]

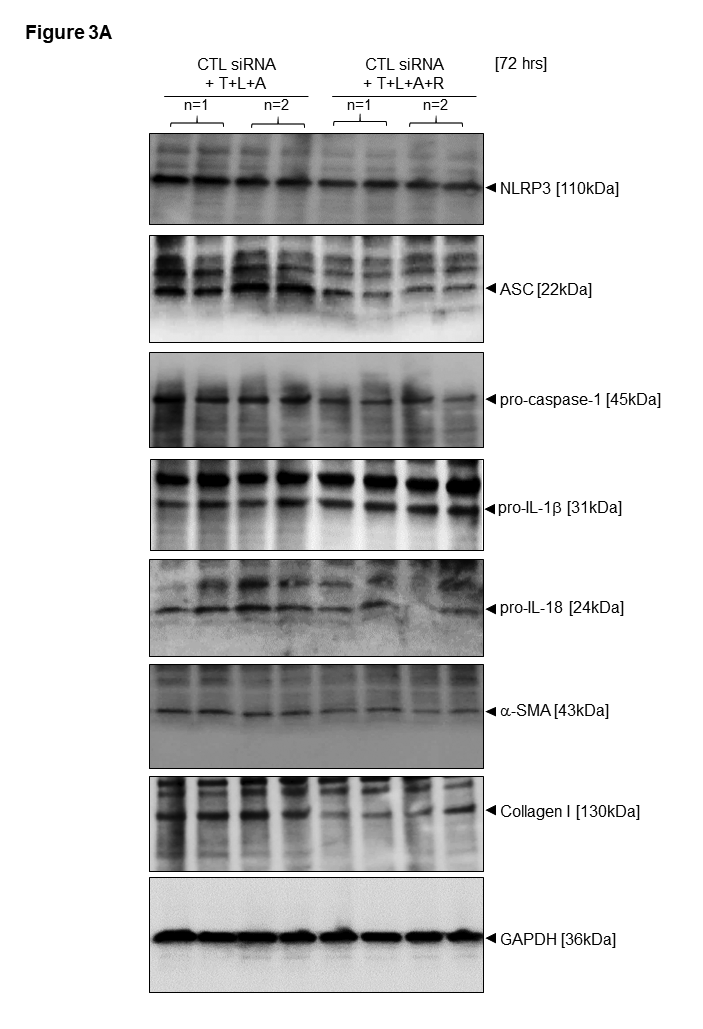

Supplement: Supplementary file 1 [file DataSheet_1.zip › Suppl_Figure 3A.tif]

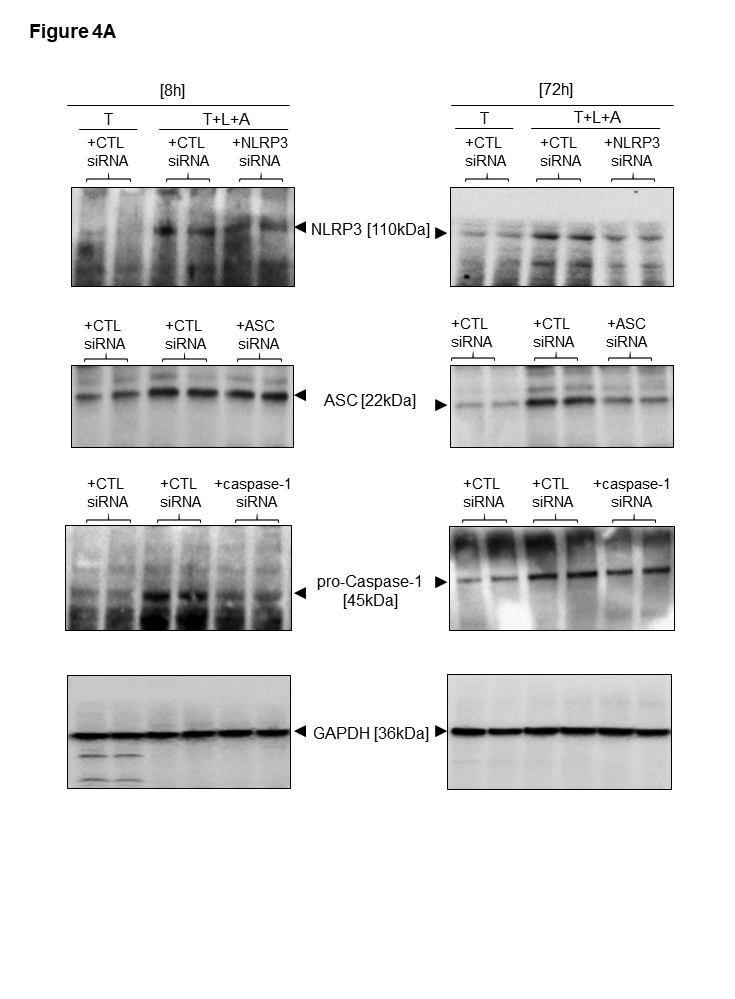

Supplement: Supplementary file 1 [file DataSheet_1.zip › Suppl_Figure 4A.tif]

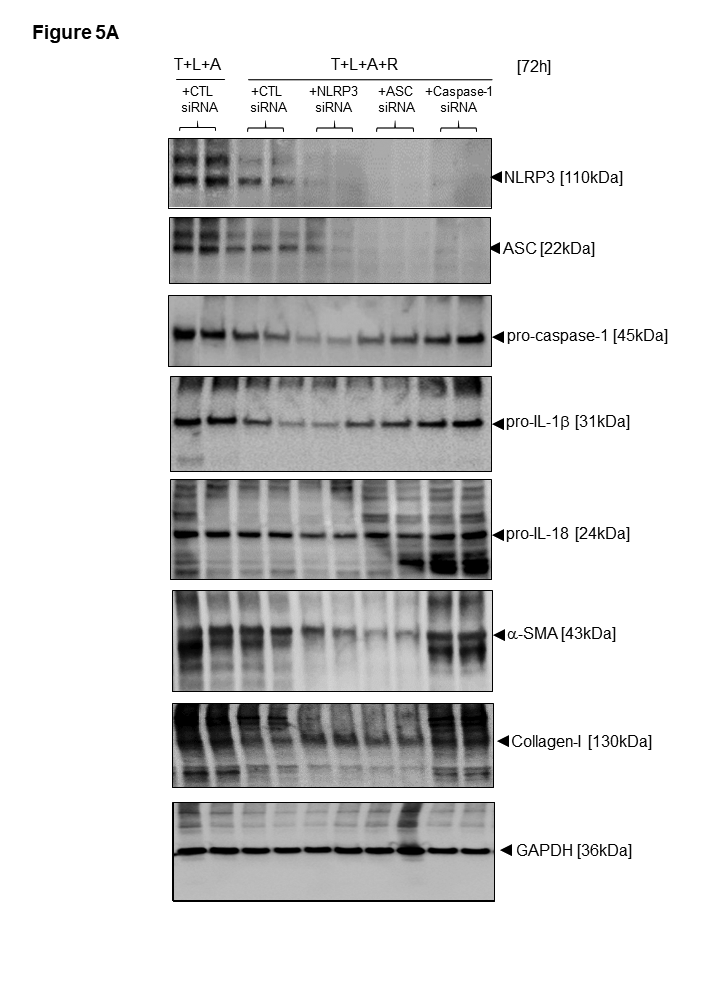

Supplement: Supplementary file 1 [file DataSheet_1.zip › Suppl_Figure 5A.tif]
