# Supplementary material for: Relaxin Can Mediate Its Anti-Fibrotic Effects by Targeting the Myofibroblast NLRP3 Inflammasome at the Level of Caspase-1
Source: Front Pharmacol. 2020 Aug 4;11:1201. doi: 10.3389/fphar.2020.01201 (PMC7417934; doi:10.3389/fphar.2020.01201)
Supplement: Supplementary file 2 [file Table_1.docx]

**Supplementary Table 1. Changes in NLRP3 inflammasome components in L+A-stimulated HDFs stimulated vs T alone- vs T+L+A-stimulated HDFs**

|  | **Treatments** | | |
| --- | --- | --- | --- |
| **NLRP3 inflammasome components** | **L+A (without T)**  **8h 72h** | **T alone**  **8h 72h** | **T+L+A**  **8h 72h** |
| NLRP3 | − − | − − | ↑ ↑ |
| ASC | − − | − − | ↑ ↑ |
| pro-caspase-1 | − − | − ↑ | ↑ ↑ |

All data are compared to respective measurements observed from unstimulated cells. ‘−’ denotes no difference in expression at the time-point indicated; ‘↑’ denotes an increased expression at the time-point indicated.
